# Infant mortality related to congenital heart disease in the United States, 1999–2025: trends, disparities, and projections to 2040

**DOI:** 10.1186/s13019-026-04533-8

**Published:** 2026-11-12

**Authors:** Amr Ibrahim, Alaa Eldeeb, Raveen Mujeeb, Alyaa Ahmed Ibrahim, Mohamed Elfeki, Mahmoud Tablawy, Krish Patel, Karim Othman Abdelnaim, Fizzah Ikram Ul Haq, Mohammed Elshiekh, Mohamed Fawzi Hemida

**Affiliations:** 1 Faculty of Medicine, Monoufia University, Monoufia, Egypt; 2 Faculty of Medicine, Alexandria University, Alexandria, Egypt; 3 Dow University of Health Sciences, Jamshoro, Pakistan; 4 Hamad Medical Corporation, Doha, Qatar; 5 Faculty of Medicine, Al-azhar University, Cairo, Egypt; 6 C. U. Shah Medical College, Surendranagar, Gujarat India; 7 Faculty of Medicine, Ain Shams University, Cairo, Egypt; 8 Allama Iqbal Medical College, Lahore, Pakistan; 9 American Society for Inclusion, Diversity, and Equity in Healthcare (ASIDE), Delaware, USA; 10 University of Vermont, Vermont, USA

**Keywords:** Congenital Heart Disease (CHD), Infant Mortality, Racial Disparities, Trend Analysis, Time-Series Forecasting

## Abstract

**Introduction:**

Congenital heart disease remains a leading congenital cause of infant mortality in the United States despite major advances in diagnosis and neonatal care. This study evaluated national mortality trends from 1999 to 2025, examined sex and racial disparities, and projected future burden through 2040 using time-series forecasting.

**Methods:**

A retrospective CDC WONDER analysis examined U.S. infant (< 1 year) CHD mortality from 1999 to 2025 using ICD-10 codes Q20–Q26. Deaths listing CHD as underlying or contributing cause were included. Crude mortality rates (CMRs) per 100,000 with 95% CIs were calculated. Trends were evaluated using Joinpoint regression analysis to estimate annual percent change (APC) and average annual percent change (AAPC). Forecasts to 2040 used ARIMA and ETS models, stratified by sex and race/ethnicity.

**Results:**

From 1999 to 2025, 55,719 infant deaths related to CHD were recorded in the United States, 92.16% in medical facilities. The CMRs declined from 69.66 in 1999 to 46.44 in 2025 (AAPC − 1.32%) and is projected at 37.51 by 2040. Male mortality remained higher than female (54.86 vs. 52.04). NH Black infants had the highest mean mortality (71.87), while NH Asian/Pacific Islander infants showed the steepest decline (AAPC − 2.30%). Recent increases appeared in NH White infants after 2018 (APC 1.56%) and Hispanic infants after 2020 (APC 3.48%) overall.

**Conclusion:**

Infant CHD related mortality declined substantially from 1999 to 2025 and is projected to continue decreasing through 2040; however, persistent excess mortality in male, NH Black, and Hispanic infants underscores enduring demographic disparities in outcomes.

**Supplementary Information:**

The online version contains supplementary material available at https://doi.org/10.1186/s13019-026-04533-8.

## Introduction

Congenital heart disease (CHD) is a structural abnormality of the heart and/or great vessels occurring at birth that results in a series of short- and long‐term adverse outcomes [1]. In the United States, heart defects affect nearly 1% of births, or about 40,000 babies, each year [2, 3]. Approximately 50% of CHD cases can be attributed to known factors, including genetic syndromes, teratogenic exposures, and maternal conditions such as diabetes. However, substantial uncertainty remains regarding the risk factors underlying the remaining 50% of cases [1, 4].

Compared with the general population, Infants with CHD, particularly those with critical congenital heart defects, are at increased risk of severe complications including hemodynamic instability, hypoxemia, heart failure, respiratory distress, and cardiogenic shock, all of which may contribute to early mortality if not promptly recognized and managed [5].

Despite some improvements in the past two decades, neonates in low-income and middle-income countries (LMICs) with severe forms of congenital heart disease and without access to surgical treatment are more likely to die before their fifth birthday than are those in high income countries (HICs) [6–8]. Although survival outcomes for infants with congenital heart disease have improved substantially in high-income countries, important disparities in mortality persist across demographic groups, highlighting ongoing challenges in achieving equitable health outcomes [7–9].

Although recent studies have examined national CHD mortality trends across all age groups, infant mortality remains insufficiently characterized despite accounting for the highest CHD-related mortality burden. Infant-specific analyses are needed because mortality patterns, risk factors, and healthcare needs differ substantially from those of older children and adults [9]. Furthermore, forecasting future mortality trends may help inform healthcare planning, resource allocation, and targeted public health interventions aimed at reducing the burden of CHD-associated infant mortality. This study aims to evaluate temporal trends in infant mortality related to CHD in the United States using nationwide mortality data from the Centers for Disease Control and Prevention (CDC WONDER Database), assess disparities across demographic subgroups, and project future mortality patterns through 2040. The CDC WONDER database serves as a valuable public health resource by providing nationwide mortality data with detailed demographic information, enabling the assessment of temporal trends and disparities across population subgroups.

## Methods

### Study design

We conducted a retrospective analysis using death certificate data obtained from the CDC WONDER database [10]. Data for infants aged < 1 between January 1999 and December 2025 was analyzed to assess mortality from CHD in the U.S. The International Statistical Classification of Diseases and Related Health Problems-10th Revision (ICD-10) was used as follows: Q20-Q26 for CHD. Those codes were used by another published study [9]. Deaths were included if CHD was listed anywhere on the death certificate, either as an underlying cause or contributing cause of death. This comprehensive approach ensures the capture of all deaths where CHD played a documented role, regardless of its position on the death certificate. Mortality data for 1999–2024 were obtained from the CDC WONDER Final Multiple Cause of Death database, whereas mortality data for 2025 were obtained from the CDC WONDER Provisional Mortality database. Institutional review board approval was not required for this study as it used de-identified public use data provided by the government and adhered to the STROBE (Strengthening the Reporting of Observational Studies in Epidemiology) guidelines for reporting [11]. In accordance with CDC-WONDER database policies, death counts < 10 are suppressed to protect confidentiality. Consequently, suppressed data were treated as missing and excluded from analysis.

### Data abstraction

Data for population size, number of deaths, and demographics such as sex and race, and place of death was extracted. Place of death was categorized into medical facilities, hospice, home and nursing home/long-term care facilities. Racial and ethnic categories were classified as non-Hispanic (NH) Black or African American, NH White, NH American Indian or Alaskan Native, NH Asian or Pacific Islander, and Hispanic or Latino.

### Statistical analysis

Crude mortality rates (CMRs) per 100,000 population from 1999 to 2025 by year, sex, and race with 95% CIs were calculated. The Joinpoint Regression Program (Joinpoint V 5.4.0.0, National Cancer Institute) was used to determine the average annual percent change (AAPC) and the annual percent change (APC) with 95% CI in CMR to quantify national annual trends in CHD related mortality. This method was used in a previous published paper with the same study design [12]. Joinpoint regression, a segmented regression technique, was used to identify points of trend change (join points) by fitting log-linear models and it used permutation tests to select the optimal number of join points, ensuring model fit [13]. APCs were considered increasing or decreasing if the slope describing the change in mortality was significantly different from zero using two tailed t testing. A value of *p* < 0.05 was considered statistically significant.

### Time series projection

To ensure the highest level of predictive precision, we evaluated the performance of two distinct forecasting approaches: the Auto-Regressive Integrated Moving Average (ARIMA) and the Error, Trend, and Seasonality (ETS) models. These models were selected a priori due to their established performance in epidemiological and demographic forecasting, and because they represent fundamentally different approaches to time-series structure: ARIMA captures autocorrelation and stochastic trends through differencing and lagged relationships, whereas ETS models explicitly decompose the series into error, trend, and seasonal components. The selection process involved comparing accuracy metrics for each demographic variable including Root Mean Square Error (RMSE), Mean Absolute Error (MAE), and Mean Absolute Percentage Error (MAPE) and analyzing the Ljung-Box residuals to identify the model that produced the most randomly distributed errors, indicating a complete capture of the underlying data patterns.

Model comparison was performed within each time series independently to ensure optimal fit for each demographic subgroup, rather than applying a single global model across heterogeneous populations. This approach reflects the fact that each sex- and race-specific group represents an independent temporal process with distinct underlying dynamics; therefore, model performance and selection were evaluated separately for each subgroup to maximize within-series predictive accuracy.

In sex stratified trends the ARIMA model was chosen as the primary projection tool. Taking the overall trends as an example, the ARIMA model achieved RMSE of 1.898 and MAE of 1.410, which demonstrated better accuracy than ETS model’s RMSE of 2.091 and MAE of 1.567.

Regarding racial demographics, the optimal model varied by group. For the Hispanic population, the Error, Trend, and Seasonality (ETS) model was selected due to a significantly lower RMSE of 3.085, compared to the ARIMA model’s RMSE of 3.715. Similarly, for the NH Asian or Pacific Islander group, the ETS model was preferred, as it achieved an RMSE of 4.343 and a Mean Absolute Scaled Error (MASE) of 0.642, which was more accurate than the ARIMA values of 5.331 and 0.682, respectively. In contrast, the ARIMA model was used for the NH Black demographic because it produced an RMSE of 4.974 and an MAE of 4.200, showing higher precision than the ETS model’s RMSE of 5.327 and MAE of 4.352. Finally, for the NH White population, the ARIMA model was chosen for the projection, delivering the lowest overall error rates with an RMSE of 1.878 and a MAPE of 2.88%, compared to the ETS model’s RMSE of 2.073 and MAPE of 3.29%.

## Results

From 1999 to 2025, 55,719 CHD-associated infant deaths were recorded in the United States. The majority of deaths occurred in medical facilities (92.16%), followed by decedent’s home (6.23%), hospice facilities (0.43%), nursing homes or long-term care facilities (0.17%), other locations (0.73%), and for 0.28%, the place of death was unknown. [Supplementary Table 3]

### Overall trends

Over the study period, the overall CMR decreased from 69.66 (95% CI: 67.00 to 72.31) in 1999 to 46.44 (95% CI: 44.14 to 48.74) in 2025, with an AAPC of -1.32 (95% CI: -1.73 to -0.91; *p* < 0.001). The mean CMR was 53.48 (95% CI: 51.17 to 55.79). The projected mean CMR is anticipated to fall to 37.51 (95% CI: 25.60 to 49.41) by 2040.

**Fig. 1:**
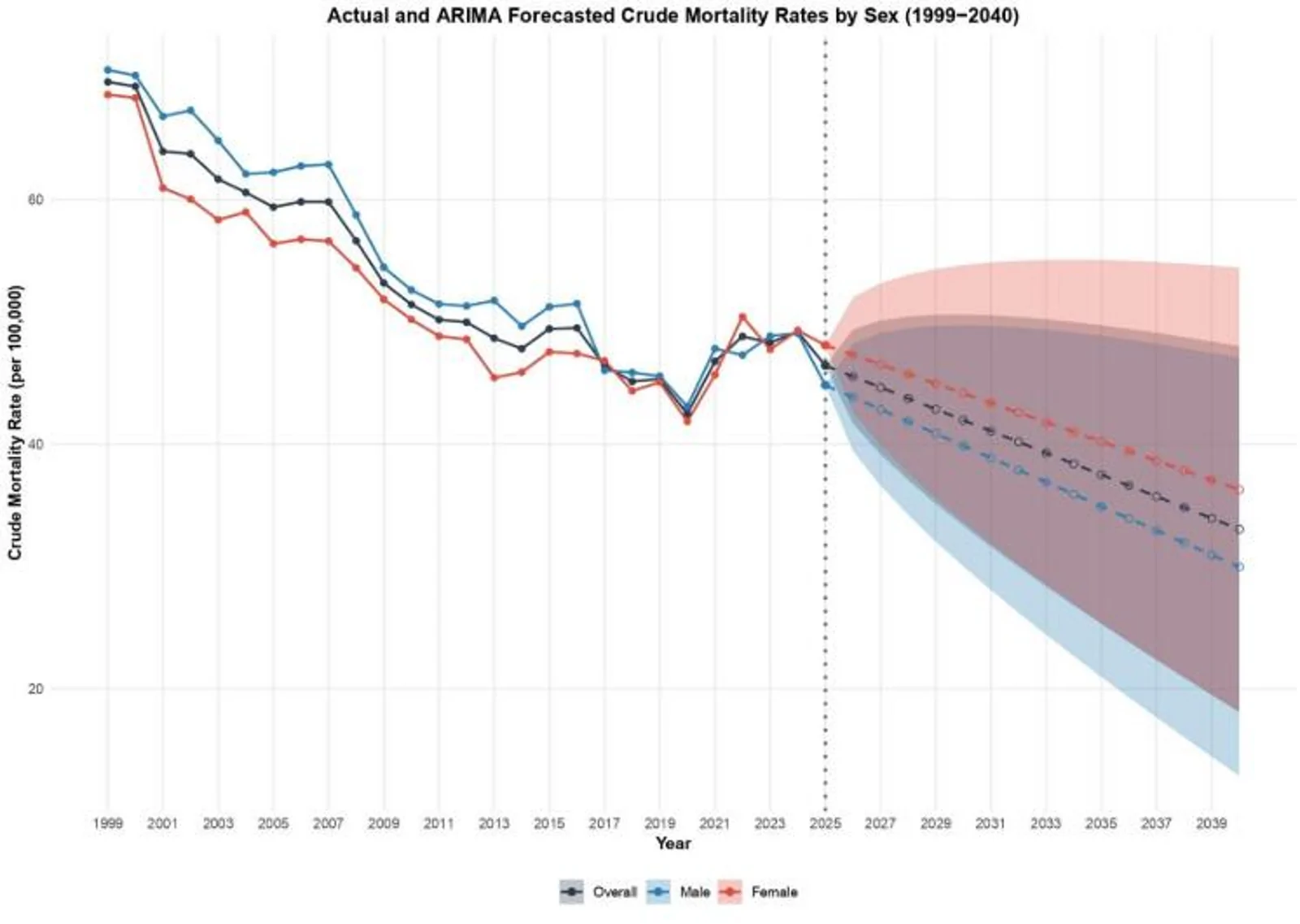
Analysis of Crude Mortality Rates by Overall and Sex: Historical Trends and ARIMA Forecasts. (1999–2040)

The CMR trend demonstrated a significant decline from 1999 to 2018 (APC: -2.23; 95% CI: -2.50 to -1.96; *p* < 0.001). This was followed by a non-significant increase from 2018 to 2025 (APC: 1.21; 95% CI: -0.26 to 2.69; *p* = 0.101). Projections suggest that the overall CMR for congenital heart disease in infants will decline to 31.26 (95% CI: 15.31 to 47.20) by 2040. [Figure 1], [Supplementary Tables 1, 4]

### Sex-stratified trends

During the study period, 29,251 deaths were reported among male infants and 26,468 among female infants. Mortality was consistently higher in male infants. Among male infants, the mean CMR was 54.86 (95% CI: 51.60 to 58.13), while among female infants it was 52.04 (95% CI: 48.78 to 55.29). An overall declining trend was observed in both male and female infants from 1999 to 2025 [Males: AAPC: -1.51 (95% CI: -2.00 to -1.00; *p* < 0.001); Females: AAPC: -1.29 (95% CI: -1.69 to -0.88; *p* < 0.001)].

Among male infants, the trend showed a significant decline from 1999 to 2019 (APC: -2.24; 95% CI: -2.52 to -1.96; *p* < 0.001), followed by a non-significant increase from 2019 to 2025 (APC: 1.01; 95% CI: -1.10 to 3.17; *p* = 0.333). Among female infants, the trend significantly decreased from 1999 to 2014 (APC: -2.55; 95% CI: -2.99 to -2.10; *p* < 0.001), followed by a non-significant increase from 2014 to 2025 (APC: 0.45; 95% CI: -0.36 to 1.27; *p* = 0.261). Forecasts suggest that the CMR is expected to reach 34.70 (95% CI: 15.33 to 54.06) among female infants, and 27.97 (95% CI: 9.77 to 46.17) among male infants by 2040. [Figure 1], [Supplementary Tables 1, 4]

### Race/Ethnicity trends

From 1999 to 2025, the highest mean CMR was observed among NH Black infants (71.87; 95% CI: 65.01 to 78.73), followed by Hispanics (53.32; 95% CI: 48.72 to 57.92), NH Whites (49.55; 95% CI: 46.50 to 52.59), and NH Asian or Pacific Islanders (38.98; 95% CI: 30.82 to 48.51). However, the greatest decrement was experienced by the Asian or Pacific Islander infants (AAPC: -2.30; 95% CI: -2.86 to -1.74; *p* < 0.001), followed by NH Blacks (AAPC: -1.68; 95% CI: -2.36 to -0.99; *p* < 0.001), NH Whites (AAPC: -1.34; 95% CI: -1.73 to -0.94; *p* < 0.001), and Hispanics (AAPC: -0.91; 95% CI: -1.62 to -0.19; *p* = 0.013).

Among Hispanic infants, the CMR trend showed a significant decline from 1999 to 2020 (APC: -1.93; 95% CI: -2.30 to -1.55; *p* < 0.001), followed by a non-significant increase from 2020 to 2025 (APC: 3.48; 95% CI: -0.18 to 7.28; *p* = 0.062). Among NH Asian or Pacific Islander infants, the CMR decreased steadily and significantly from 1999 to 2025 (APC: -2.30; 95% CI: -2.86 to -1.74; *p* < 0.001). Among NH Black infants, the trend showed a significant decline from 1999 to 2013 (APC: -2.58; 95% CI: -3.41 to -1.74; *p* < 0.001), followed by a non-significant decline from 2013 to 2025 (APC: -0.61; 95% CI: -1.84 to 0.63; *p* = 0.319). Among NH White infants, the CMR showed a significant decline from 1999 to 2018 (APC: -2.39; 95% CI: -2.64 to -2.14; *p* < 0.001), followed by a significant increase from 2018 to 2025 (APC: 1.56; 95% CI: 0.14 to 3.00; *p* = 0.033).

**Fig. 2:**
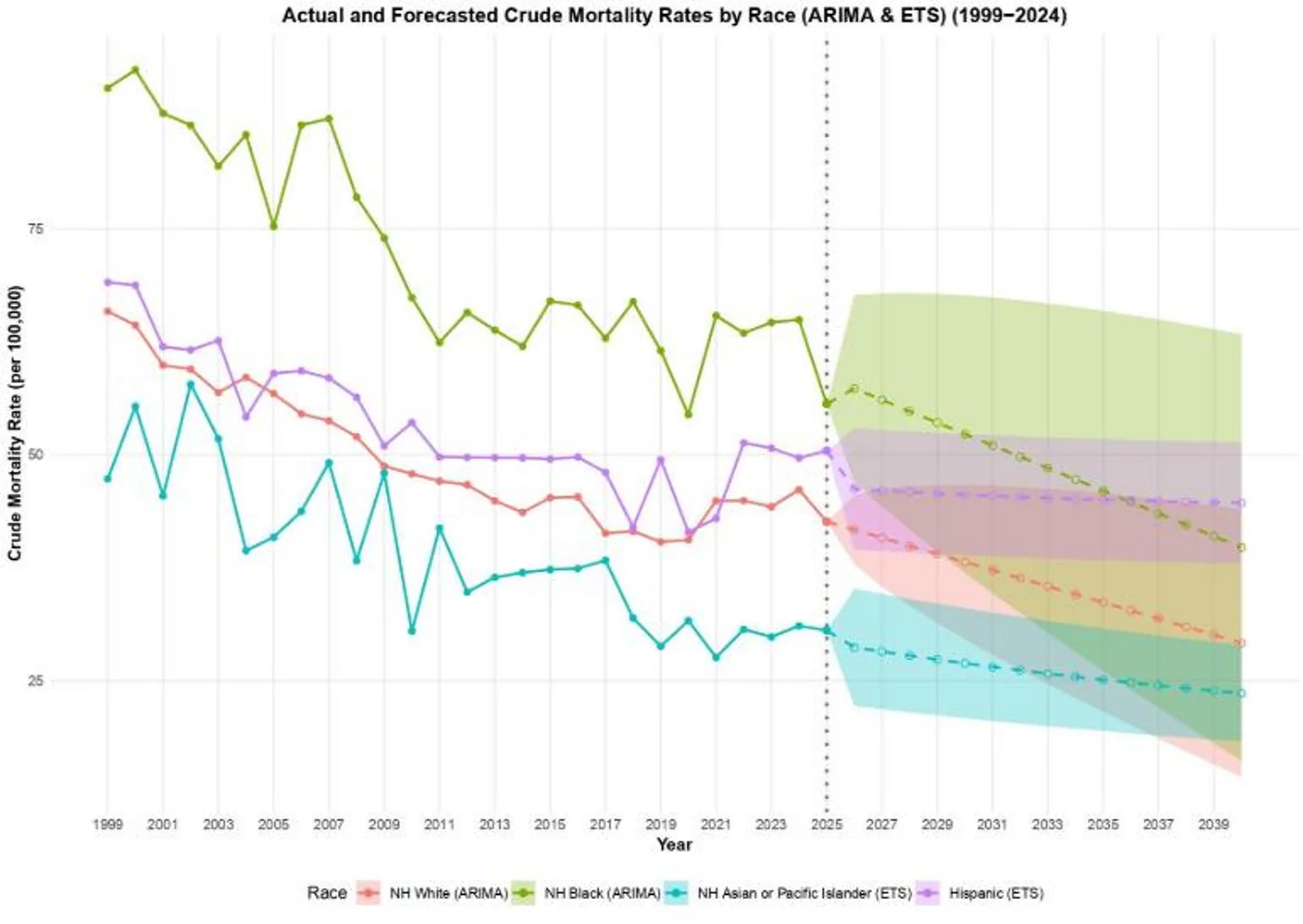
Analysis of Crude Mortality Rates by Sex: Historical Trends and Forecasts (ARIMA and ETS). (1999–2040)

The forecasted CMR is expected to reach 44.55 (95% CI: 37.85 to 51.25) among Hispanics, 23.08 (95% ci: 17.86 to 28.30) among Asian or Pacific Islanders, 37.21 (95% CI: 12.28 to 62.14) among NH Black infants, and 27.38 (95% CI: 11.60 to 43.15) among NH Whites. [Figure 2], [Supplementary Tables 2, 5]

## Discussion

This national trend analysis of infant mortality related to CHD in the United States from 1999 to 2025 demonstrated a substantial long-term reduction in CMRs, with projections suggesting continued decline through 2040. The steepest reduction occurred between 1999 and 2018, followed by a recent plateau with a non-significant upward inflection after 2018. Mortality remained consistently higher among male infants than female infants across the study period, despite significant declines in both groups. Pronounced racial and ethnic disparities persisted, with NH Black infants bearing the highest mortality burden, while NH Asian or Pacific Islander infants experienced the most rapid decline. The differing mortality patterns observed across racial and ethnic groups underscore the importance of continued monitoring of demographic disparities in CHD-associated infant mortality.

The marked decline in infant CHD mortality is consistent with major advances in prenatal diagnosis, universal newborn screening for critical CHD, neonatal transport systems, lesion-specific surgical strategies, and postoperative intensive care. The widespread adoption of pulse oximetry screening after its inclusion in U.S. newborn screening recommendations substantially improved early recognition of ductal-dependent lesions and reduced preventable circulatory collapse, contributing to lower early infant mortality [14–16]. Additional survival gains likely reflect improved fetal echocardiographic detection, earlier planned delivery at tertiary cardiac centers, and refinements in neonatal surgical palliation pathways for high-risk lesions such as hypoplastic left heart syndrome and transposition physiology. Large epidemiologic studies have similarly documented sustained reductions in CHD-related childhood mortality over recent decades, supporting the biologic plausibility of the observed downward trend [17, 18].

The attenuation of mortality decline after approximately 2018 likely reflects a shift from technology-driven improvements toward residual deaths increasingly determined by health-system performance and social risk. Maternal health trends, including increasing rates of obesity and pre-gestational diabetes, may also contribute to the incidence and complexity of CHD and could represent an additional factor influencing recent mortality patterns [1, 4]. Contemporary pediatric CHD literature suggests that once major perioperative advances mature, remaining mortality becomes disproportionately influenced by interstage monitoring, rescue escalation systems, post-discharge continuity, and socioeconomic barriers to rapid re-evaluation [19, 20].

The predominance of deaths occurring in medical facilities in this cohort reinforces the importance of inpatient deterioration pathways, perioperative complications, and delayed recognition of decompensation as major remaining contributors. In addition, increasing survival of infants born with more complex lesions may paradoxically preserve a pool of medically fragile survivors at persistent risk of first-year mortality, potentially contributing to the recent flattening in national rates [16–19]. This pattern may reflect shifting mortality profiles and competing risks, whereby improvements in early survival alter the timing of mortality within the first year of life rather than completely eliminating mortality risk.

The persistently higher mortality observed among male infants aligns with prior national mortality studies showing sex-based disparities in CHD outcomes, with male sex associated with higher mortality across several pediatric age groups and lesion categories [17]. This difference may be partially explained by sex-linked variation in the prevalence of severe structural lesions, extracardiac comorbidity patterns, and differential vulnerability to perioperative complications [17]. Importantly, both sexes demonstrated significant long-term improvement, suggesting that advances in detection and neonatal cardiac care have been broadly effective. Nevertheless, the persistence of higher male mortality despite similar trajectories indicates that biologic susceptibility and lesion distribution likely continue to influence infant outcomes independent of secular improvements in care delivery. However, the forecasting analyses suggest a gradual convergence of sex-specific mortality rates over time, and any projected crossover should be interpreted cautiously, as it reflects model-based extrapolation rather than an established biological mechanism.

The racial and ethnic findings remain among the most clinically significant aspects of this analysis. NH Black infants experienced the highest CHD mortality burden across the entire study period, consistent with longstanding U.S. evidence demonstrating persistent racial disparities in both infant survival and postoperative congenital cardiac outcomes [21–23]. These disparities are unlikely to be explained solely by lesion prevalence and instead strongly suggest the cumulative effects of structural inequities in prenatal diagnosis, maternal-fetal referral pathways, insurance continuity, access to high-volume surgical centers, and longitudinal pediatric cardiology follow-up. The slower improvement among Hispanic infants and the plateauing decline among Black infants further support the role of systemic barriers that extend beyond the neonatal hospitalization. By contrast, the steepest decline among NH Asian or Pacific Islander infants may reflect differential access to specialized perinatal cardiac services and more favorable healthcare utilization patterns, although residual heterogeneity within this broad category remains important [20–23]. These findings underscore the need for targeted interventions aimed at improving equitable access to prenatal detection, specialized cardiac care, and longitudinal follow-up among historically underserved populations.

The projected continuation of mortality decline through 2040 is encouraging, but subgroup-specific forecasts suggest that demographic inequities are likely to persist despite lower national averages. This pattern emphasizes that future reductions in infant CHD mortality may depend less on new operative breakthroughs and more on equitable implementation of prenatal screening, regionalization of complex neonatal cardiac surgery, telecardiology-supported surveillance, and standardized interstage home-monitoring pathways. Evidence from recent disparity-focused pediatric CHD frameworks increasingly supports system-level interventions targeting structural racism, geographic inequity, and care fragmentation as the most promising route to further mortality reduction [20, 23, 24]. These findings further underscore the importance of sustained investment in CHD surveillance, research, and public health programs to monitor evolving trends, evaluate disparities, and guide evidence-based policy and healthcare planning.

## Strengths

This study has several notable strengths. It leverages a large, nationally representative dataset spanning more than two decades, allowing for robust assessment of long-term mortality trends in infant CHD across the United States. The use of multiple-cause-of-death data enhances sensitivity by capturing all deaths in which CHD contributed, rather than restricting analyses to the underlying cause alone. Additionally, the application of Joinpoint regression provides a rigorous evaluation of temporal changes, identifying inflection points in mortality trends, while the incorporation of forecasting models (ARIMA and ETS) offers valuable insight into future mortality patterns. The stratified analyses by sex and race/ethnicity further strengthen the study by highlighting important demographic disparities that may inform targeted public health and clinical interventions.

## Limitations

Several limitations should be acknowledged. First, this is a retrospective analysis based on death certificate data, which is subject to misclassification and coding inaccuracies. The use of ICD-10 codes does not allow for validation of clinical diagnoses, nor does it capture lesion-specific details, disease severity, or timing of diagnosis. Additionally, multiple-cause-of-death data do not establish temporal or causal relationships, making it impossible to determine whether CHD was the primary driver of mortality or a contributing condition. Important clinical variables, such as surgical interventions, postoperative complications, prenatal diagnosis, and comorbid conditions, are not available in the dataset, limiting the ability to contextualize observed mortality patterns.

Second, the analysis is constrained by limitations inherent to aggregated population-level data. Suppression of death counts fewer than 10, as required by database policy, may introduce bias, particularly in subgroup analyses involving smaller racial or ethnic populations. While temporal trends were rigorously assessed for mortality rates, variables such as place of death were analyzed only as aggregate distributions across the study period, without temporal stratification, which limits interpretation of changes over time. Furthermore, projections using ARIMA and ETS models are dependent on historical patterns and assumptions of trend continuity, and therefore may not account for future changes in healthcare delivery, technological advances, or policy interventions that could significantly alter mortality trajectories.

Third, mortality data for 2025 were obtained from the CDC WONDER Provisional Mortality Database and may be subject to future revision. Therefore, the most recent mortality estimates should be interpreted with caution until finalized data become available.

## Conclusion

Infant mortality related to CHD in the United States declined significantly between 1999 and 2025, with an overall reduction in CMRs and projections indicating continued decreases through 2040. The decline was most pronounced prior to 2018, followed by a recent plateau with a non-significant upward trend. Mortality rates remained consistently higher among male infants compared to females, although both groups experienced significant long-term reductions. Racial and ethnic disparities persisted throughout the study period, with NH Black infants exhibiting the highest mortality rates, while NH Asian or Pacific Islander infants demonstrated the greatest rate of decline. The majority of deaths occurred in medical facilities, with smaller proportions occurring at home and other settings. These findings highlight sustained improvements in CHD-related infant mortality alongside persistent demographic disparities and recent slowing in the pace of decline.

## Supplementary Information

Supplementary Material 1

## Data Availability

The data that support the findings of this study are openly available in CDC‐WONDER at https://wonder.cdc.gov/. The data supporting the findings of this study were obtained from the CDC WONDER online database (Centers for Disease Control and Prevention Wide‐ranging Online Data for Epidemiologic Research). Further inquiries can be directed to the corresponding author.
