# Supplementary material for: Infant mortality related to congenital heart disease in the United States, 1999–2025: trends, disparities, and projections to 2040
Source: J Cardiothorac Surg. 2026 Nov 12;21:886. doi: 10.1186/s13019-026-04533-8 (PMC13756874; doi:10.1186/s13019-026-04533-8)
Supplement: Supplementary file 1 [file 13019_2026_4533_MOESM1_ESM.docx]

**Supplemental Table 1:** Overall and sex-stratified trends in Infant Mortality from Congenital Heart Disease, shown as crude rates in the United States, 1999–2025.

| **Year** | **Crude Rate (95% Confidence Interval** | | |
| --- | --- | --- | --- |
|  | **Overall** | **Female** | **Male** |
| 1999 | 69.66 (67 - 72.31) | 68.62 (64.85 - 72.4) | 70.64 (66.9 - 74.38) |
| 2000 | 69.29 (66.65 - 71.94) | 68.35 (64.59 - 72.11) | 70.19 (66.47 - 73.91) |
| 2001 | 63.97 (61.5 - 66.45) | 60.98 (57.53 - 64.44) | 66.84 (63.3 - 70.38) |
| 2002 | 63.77 (61.28 - 66.26) | 60.06 (56.61 - 63.52) | 67.33 (63.75 - 70.91) |
| 2003 | 61.7 (59.26 - 64.14) | 58.37 (54.98 - 61.77) | 64.87 (61.37 - 68.37) |
| 2004 | 60.61 (58.2 - 63.02) | 59.01 (55.62 - 62.41) | 62.13 (58.72 - 65.54) |
| 2005 | 59.41 (57.02 - 61.8) | 56.42 (53.09 - 59.75) | 62.26 (58.85 - 65.68) |
| 2006 | 59.85 (57.47 - 62.24) | 56.78 (53.46 - 60.11) | 62.78 (59.37 - 66.2) |
| 2007 | 59.84 (57.48 - 62.19) | 56.63 (53.35 - 59.91) | 62.9 (59.52 - 66.28) |
| 2008 | 56.65 (54.35 - 58.94) | 54.43 (51.21 - 57.64) | 58.77 (55.5 - 62.04) |
| 2009 | 53.2 (50.94 - 55.46) | 51.86 (48.67 - 55.05) | 54.49 (51.29 - 57.69) |
| 2010 | 51.44 (49.2 - 53.68) | 50.21 (47.05 - 53.37) | 52.62 (49.46 - 55.79) |
| 2011 | 50.19 (48 - 52.39) | 48.85 (45.75 - 51.95) | 51.48 (48.36 - 54.59) |
| 2012 | 49.99 (47.78 - 52.19) | 48.59 (45.48 - 51.7) | 51.32 (48.19 - 54.45) |
| 2013 | 48.68 (46.51 - 50.86) | 45.45 (42.44 - 48.46) | 51.77 (48.63 - 54.91) |
| 2014 | 47.82 (45.66 - 49.97) | 45.9 (42.87 - 48.92) | 49.66 (46.58 - 52.73) |
| 2015 | 49.45 (47.26 - 51.63) | 47.56 (44.49 - 50.62) | 51.25 (48.14 - 54.36) |
| 2016 | 49.52 (47.33 - 51.71) | 47.43 (44.37 - 50.5) | 51.51 (48.39 - 54.64) |
| 2017 | 46.43 (44.3 - 48.56) | 46.83 (43.77 - 49.88) | 46.05 (43.09 - 49.01) |
| 2018 | 45.14 (43.02 - 47.26) | 44.37 (41.36 - 47.38) | 45.87 (42.88 - 48.86) |
| 2019 | 45.33 (43.19 - 47.48) | 45.08 (42.02 - 48.14) | 45.58 (42.57 - 48.59) |
| 2020 | 42.49 (40.4 - 44.58) | 41.87 (38.91 - 44.84) | 43.08 (40.13 - 46.02) |
| 2021 | 46.78 (44.46 - 49.11) | 45.69 (42.41 - 48.98) | 47.83 (44.54 - 51.11) |
| 2022 | 48.83 (46.5 - 51.17) | 50.44 (47.05 - 53.84) | 47.3 (44.08 - 50.51) |
| 2023 | 48.32 (45.98 - 50.65) | 47.76 (44.43 - 51.08) | 48.86 (45.57 - 52.14) |
| 2024 | 49.2 (46.83 - 51.57) | 49.32 (45.93 - 52.71) | 49.08 (45.77 - 52.39) |
| 2025 | 46.44 (44.14 - 48.74) | 48.11 (44.76 - 51.46) | 44.84 (41.68 - 48.01) |

**Supplemental Table 2:** Race-stratified trends in Infant Mortality from Congenital Heart Disease, shown as crude rate in the United States, 1999–2025.

| **Year** | **Crude Rate (95% Confidence Interval** | | | |
| --- | --- | --- | --- | --- |
|  | **Hispanic** | **NH Asian** | **NH Black** | **NH White** |
| 1999 | 69.07 (63.15 - 75) | 47.32 (36.96 - 59.68) | 90.55 (82.73 - 98.38) | 65.86 (62.54 - 69.19) |
| 2000 | 68.74 (62.89 - 74.59) | 55.34 (44.2 - 68.43) | 92.57 (84.72 - 100.42) | 64.32 (61.01 - 67.62) |
| 2001 | 61.94 (56.72 - 67.17) | 45.44 (36.14 - 56.41) | 87.77 (80.29 - 95.24) | 59.89 (56.74 - 63.05) |
| 2002 | 61.58 (56.42 - 66.74) | 57.78 (46.68 - 68.89) | 86.45 (78.93 - 93.97) | 59.45 (56.27 - 62.63) |
| 2003 | 62.61 (57.48 - 67.73) | 51.75 (42.01 - 63.06) | 81.91 (74.52 - 89.29) | 56.87 (53.76 - 59.99) |
| 2004 | 54.17 (49.48 - 58.85) | 39.36 (31.01 - 49.27) | 85.4 (77.89 - 92.92) | 58.55 (55.39 - 61.71) |
| 2005 | 58.98 (54.18 - 63.78) | 40.86 (32.4 - 50.86) | 75.25 (68.2 - 82.3) | 56.76 (53.62 - 59.91) |
| 2006 | 59.27 (54.54 - 64.01) | 43.72 (34.97 - 54) | 86.46 (78.98 - 93.93) | 54.52 (51.43 - 57.61) |
| 2007 | 58.48 (53.89 - 63.08) | 49.1 (39.52 - 58.67) | 87.17 (79.85 - 94.48) | 53.74 (50.68 - 56.8) |
| 2008 | 56.34 (51.83 - 60.85) | 38.26 (30.43 - 47.49) | 78.49 (71.56 - 85.43) | 51.98 (48.95 - 55) |
| 2009 | 50.97 (46.61 - 55.32) | 47.96 (38.98 - 58.39) | 73.95 (67.16 - 80.75) | 48.72 (45.74 - 51.7) |
| 2010 | 53.54 (49.03 - 58.04) | 30.48 (23.37 - 39.08) | 67.37 (60.82 - 73.91) | 47.87 (44.9 - 50.84) |
| 2011 | 49.74 (45.47 - 54) | 41.87 (33.58 - 51.58) | 62.41 (56.18 - 68.64) | 47.07 (44.12 - 50.02) |
| 2012 | 49.69 (45.37 - 54.01) | 34.79 (27.27 - 43.74) | 65.73 (59.29 - 72.17) | 46.66 (43.71 - 49.62) |
| 2013 | 49.66 (45.32 - 54) | 36.42 (28.79 - 45.45) | 63.77 (57.41 - 70.12) | 44.89 (42 - 47.78) |
| 2014 | 49.64 (45.31 - 53.98) | 36.94 (29.42 - 45.8) | 62 (55.74 - 68.25) | 43.61 (40.76 - 46.46) |
| 2015 | 49.51 (45.2 - 53.82) | 37.28 (29.65 - 46.27) | 66.99 (60.53 - 73.46) | 45.23 (42.34 - 48.13) |
| 2016 | 49.74 (45.42 - 54.06) | 37.4 (29.88 - 46.25) | 66.54 (60.07 - 73.01) | 45.32 (42.42 - 48.22) |
| 2017 | 48.02 (43.79 - 52.25) | 38.3 (30.59 - 47.36) | 62.87 (56.56 - 69.19) | 41.3 (38.51 - 44.08) |
| 2018 | 41.98 (37.98 - 45.98) | 31.92 (24.83 - 40.39) | 66.92 (60.33 - 73.51) | 41.52 (38.69 - 44.34) |
| 2019 | 49.42 (45.02 - 53.81) | 28.8 (21.98 - 37.07) | 61.5 (55.12 - 67.88) | 40.35 (37.55 - 43.16) |
| 2020 | 41.43 (37.38 - 45.48) | 31.63 (24.88 - 39.65) | 54.42 (48.47 - 60.37) | 40.56 (37.7 - 43.43) |
| 2021 | 42.94 (38.65 - 47.24) | 27.56 (20.76 - 35.88) | 65.38 (58.32 - 72.45) | 44.89 (41.7 - 48.07) |
| 2022 | 51.3 (46.74 - 55.86) | 30.66 (23.66 - 39.07) | 63.45 (56.75 - 70.14) | 44.91 (41.73 - 48.09) |
| 2023 | 50.72 (46.19 - 55.24) | 29.83 (22.77 - 38.4) | 64.63 (57.72 - 71.54) | 44.22 (41.06 - 47.37) |
| 2024 | 49.63 (45.24 - 54.02) | 31.05 (23.91 - 39.65) | 64.92 (57.89 - 71.94) | 46.12 (42.84 - 49.4) |
| 2025 | 50.43 (46.01 - 54.86) | 30.56 (23.49 - 39.1) | 55.61 (49.11 - 62.12) | 42.59 (39.44 - 45.75) |

**Supplemental Table 3:**  Place of death in the United States (1999–2025) for Infant Mortality from Congenital Heart Disease.

| **Place of Death** | **Deaths** | **%** |
| --- | --- | --- |
| Medical Facility - Inpatient | 46766 | 83.94% |
| Medical Facility - Outpatient or ER | 4216 | 7.57% |
| Medical Facility - Dead on Arrival | 294 | 0.53% |
| Medical Facility - Status unknown | 80 | 0.14% |
| Decedent's home | 3469 | 6.23% |
| Hospice facility | 238 | 0.43% |
| Nursing home/long term care | 89 | 0.16% |
| Other | 409 | 0.73% |
| Place of death unknown | 155 | 0.28% |
| **Total** | **55716** | **100%** |

**Supplementary Table 4.** Forecasted Crude Mortality Rates by Overall and Sex from 2026 to 2040 Using Auto-Regressive Integrated Moving Average (ARIMA) Modeling.

| Sex | Year | Crude Rate | ARIMA forecasted Crude Rate Lower 95% Confidence Interval | ARIMA forecasted Crude Rate Upper 95% Confidence Interval |
| --- | --- | --- | --- | --- |
| Overall | 2026 | 43.76077 | 37.06277 | 50.45877 |
| Overall | 2027 | 42.86769 | 35.1335 | 50.60188 |
| Overall | 2028 | 41.97462 | 33.32753 | 50.6217 |
| Overall | 2029 | 41.08154 | 31.60913 | 50.55395 |
| Overall | 2030 | 40.18846 | 29.95709 | 50.41983 |
| Overall | 2031 | 39.29538 | 28.35759 | 50.23318 |
| Overall | 2032 | 38.40231 | 26.80102 | 50.00359 |
| Overall | 2033 | 37.50923 | 25.2804 | 49.73806 |
| Overall | 2034 | 36.61615 | 23.79045 | 49.44186 |
| Overall | 2035 | 35.72308 | 22.32707 | 49.11909 |
| Overall | 2036 | 34.83 | 20.88699 | 48.77301 |
| Overall | 2037 | 33.93692 | 19.46758 | 48.40627 |
| Overall | 2038 | 33.04385 | 18.06665 | 48.02104 |
| Overall | 2039 | 32.15077 | 16.68239 | 47.61915 |
| Overall | 2040 | 31.25769 | 15.31325 | 47.20213 |
| Female | 2026 | 45.74346 | 37.60845 | 53.87847 |
| Female | 2027 | 44.95462 | 35.56112 | 54.34811 |
| Female | 2028 | 44.16577 | 33.66352 | 54.66802 |
| Female | 2029 | 43.37692 | 31.87228 | 54.88156 |
| Female | 2030 | 42.58808 | 30.16165 | 55.01451 |
| Female | 2031 | 41.79923 | 28.51482 | 55.08364 |
| Female | 2032 | 41.01038 | 26.92014 | 55.10063 |
| Female | 2033 | 40.22154 | 25.36911 | 55.07396 |
| Female | 2034 | 39.43269 | 23.85534 | 55.01005 |
| Female | 2035 | 38.64385 | 22.37383 | 54.91386 |
| Female | 2036 | 37.855 | 20.92063 | 54.78937 |
| Female | 2037 | 37.06615 | 19.49253 | 54.63978 |
| Female | 2038 | 36.27731 | 18.08688 | 54.46774 |
| Female | 2039 | 35.48846 | 16.70147 | 54.27546 |
| Female | 2040 | 34.69962 | 15.33442 | 54.06481 |
| Male | 2026 | 41.86308 | 34.21595 | 49.5102 |
| Male | 2027 | 40.87077 | 32.04063 | 49.70091 |
| Male | 2028 | 39.87846 | 30.00606 | 49.75086 |
| Male | 2029 | 38.88615 | 28.07148 | 49.70082 |
| Male | 2030 | 37.89385 | 26.21267 | 49.57503 |
| Male | 2031 | 36.90154 | 24.41383 | 49.38924 |
| Male | 2032 | 35.90923 | 22.66402 | 49.15444 |
| Male | 2033 | 34.91692 | 20.95524 | 48.8786 |
| Male | 2034 | 33.92462 | 19.28148 | 48.56775 |
| Male | 2035 | 32.93231 | 17.63805 | 48.22656 |
| Male | 2036 | 31.94 | 16.02124 | 47.85876 |
| Male | 2037 | 30.94769 | 14.42801 | 47.46738 |
| Male | 2038 | 29.95538 | 12.85589 | 47.05488 |
| Male | 2039 | 28.96308 | 11.30279 | 46.62336 |
| Male | 2040 | 27.97077 | 9.766965 | 46.17457 |

**ARIMA;** Auto-Regressive Integrated Moving Average model

**Supplementary Table 5.** Forecasted Crude Mortality Rate by Race from 2026 to 2040 Using Auto-Regressive Integrated Moving Average (ARIMA) and Error, Trend and Seasonality (ETS) Modeling.

| Race | Year | Crude Rate | Crude Rate Lower 95% Confidence Interval | Crude Rate Upper 95% Confidence Interval | Forecast Model |
| --- | --- | --- | --- | --- | --- |
| Hispanic | 2026 | 45.85611 | 39.157 | 52.55522 | ETS |
| Hispanic | 2027 | 45.70297 | 39.00386 | 52.40208 | ETS |
| Hispanic | 2028 | 45.56238 | 38.86327 | 52.26149 | ETS |
| Hispanic | 2029 | 45.43329 | 38.73418 | 52.1324 | ETS |
| Hispanic | 2030 | 45.31477 | 38.61566 | 52.01388 | ETS |
| Hispanic | 2031 | 45.20595 | 38.50684 | 51.90506 | ETS |
| Hispanic | 2032 | 45.10604 | 38.40693 | 51.80516 | ETS |
| Hispanic | 2033 | 45.01431 | 38.3152 | 51.71343 | ETS |
| Hispanic | 2034 | 44.93009 | 38.23097 | 51.62921 | ETS |
| Hispanic | 2035 | 44.85277 | 38.15365 | 51.55189 | ETS |
| Hispanic | 2036 | 44.78177 | 38.08265 | 51.48089 | ETS |
| Hispanic | 2037 | 44.71659 | 38.01746 | 51.41571 | ETS |
| Hispanic | 2038 | 44.65674 | 37.95761 | 51.35587 | ETS |
| Hispanic | 2039 | 44.60179 | 37.90266 | 51.30093 | ETS |
| Hispanic | 2040 | 44.55135 | 37.85221 | 51.25048 | ETS |
| NH Asian or Pacific Islander | 2026 | 27.76035 | 21.48201 | 34.03869 | ETS |
| NH Asian or Pacific Islander | 2027 | 27.33013 | 21.14909 | 33.51118 | ETS |
| NH Asian or Pacific Islander | 2028 | 26.91717 | 20.82952 | 33.00482 | ETS |
| NH Asian or Pacific Islander | 2029 | 26.52078 | 20.52278 | 32.51878 | ETS |
| NH Asian or Pacific Islander | 2030 | 26.14029 | 20.22833 | 32.05224 | ETS |
| NH Asian or Pacific Islander | 2031 | 25.77506 | 19.94571 | 31.60441 | ETS |
| NH Asian or Pacific Islander | 2032 | 25.42448 | 19.67441 | 31.17454 | ETS |
| NH Asian or Pacific Islander | 2033 | 25.08796 | 19.414 | 30.76193 | ETS |
| NH Asian or Pacific Islander | 2034 | 24.76495 | 19.16404 | 30.36586 | ETS |
| NH Asian or Pacific Islander | 2035 | 24.45489 | 18.9241 | 29.98568 | ETS |
| NH Asian or Pacific Islander | 2036 | 24.15727 | 18.69379 | 29.62076 | ETS |
| NH Asian or Pacific Islander | 2037 | 23.87159 | 18.47272 | 29.27047 | ETS |
| NH Asian or Pacific Islander | 2038 | 23.59738 | 18.26051 | 28.93424 | ETS |
| NH Asian or Pacific Islander | 2039 | 23.33416 | 18.05682 | 28.6115 | ETS |
| NH Asian or Pacific Islander | 2040 | 23.0815 | 17.86129 | 28.3017 | ETS |
| NH Black | 2026 | 54.79445 | 41.70686 | 67.88205 | ARIMA |
| NH Black | 2027 | 53.53836 | 39.27466 | 67.80206 | ARIMA |
| NH Black | 2028 | 52.28226 | 36.93231 | 67.63222 | ARIMA |
| NH Black | 2029 | 51.02617 | 34.66191 | 67.39043 | ARIMA |
| NH Black | 2030 | 49.77007 | 32.4508 | 67.08934 | ARIMA |
| NH Black | 2031 | 48.51398 | 30.28968 | 66.73828 | ARIMA |
| NH Black | 2032 | 47.25788 | 28.17142 | 66.34434 | ARIMA |
| NH Black | 2033 | 46.00179 | 26.09046 | 65.91311 | ARIMA |
| NH Black | 2034 | 44.74569 | 24.04234 | 65.44905 | ARIMA |
| NH Black | 2035 | 43.4896 | 22.02342 | 64.95578 | ARIMA |
| NH Black | 2036 | 42.2335 | 20.03069 | 64.43631 | ARIMA |
| NH Black | 2037 | 40.97741 | 18.06163 | 63.89318 | ARIMA |
| NH Black | 2038 | 39.72131 | 16.11409 | 63.32853 | ARIMA |
| NH Black | 2039 | 38.46522 | 14.18624 | 62.74419 | ARIMA |
| NH Black | 2040 | 37.20912 | 12.27648 | 62.14176 | ARIMA |
| NH White | 2024 | 41.695 | 37.86921 | 45.52079 | ARIMA |
| NH White | 2025 | 40.8 | 35.38952 | 46.21048 | ARIMA |
| NH White | 2026 | 39.905 | 33.27854 | 46.53146 | ARIMA |
| NH White | 2027 | 39.01 | 31.35842 | 46.66158 | ARIMA |
| NH White | 2028 | 38.115 | 29.56028 | 46.66972 | ARIMA |
| NH White | 2029 | 37.22 | 27.84877 | 46.59123 | ARIMA |
| NH White | 2030 | 36.325 | 26.20292 | 46.44708 | ARIMA |
| NH White | 2031 | 35.43 | 24.60904 | 46.25096 | ARIMA |
| NH White | 2032 | 34.535 | 23.05764 | 46.01236 | ARIMA |
| NH White | 2033 | 33.64 | 21.5418 | 45.7382 | ARIMA |
| NH White | 2034 | 32.745 | 20.0563 | 45.4337 | ARIMA |
| NH White | 2035 | 31.85 | 18.59708 | 45.10292 | ARIMA |
| NH White | 2036 | 30.955 | 17.16093 | 44.74907 | ARIMA |
| NH White | 2037 | 30.06 | 15.74521 | 44.37479 | ARIMA |
| NH White | 2038 | 29.165 | 14.34779 | 43.98221 | ARIMA |
| NH White | 2039 | 28.27 | 12.96685 | 43.57315 | ARIMA |
| NH White | 2040 | 27.375 | 11.60087 | 43.14913 | ARIMA |

**ARIMA;** Auto-Regressive Integrated Moving Average model, **ETS;** Error, trend and seasonality model
